# Photoionisation of the tropyl radical

**DOI:** 10.3762/bjoc.9.77

**Published:** 2013-04-09

**Authors:** Kathrin H Fischer, Patrick Hemberger, Andras Bodi, Ingo Fischer

**Affiliations:** 1 Institute of Physical and Theoretical Chemistry, University of Würzburg, Am Hubland Süd, 97074 Würzburg, Germany; 2 Molecular Dynamics Group, Paul Scherrer Institut (PSI), 5232 Villigen, Switzerland

**Keywords:** dissociative photoionisation, gas phase, reactive intermediates, synchrotron radiation, threshold photoelectron spectroscopy

## Abstract

We present a study on the photoionisation of the cycloheptatrienyl (tropyl) radical, C_7_H_7_, using tunable vacuum ultraviolet synchrotron radiation. Tropyl is generated by flash pyrolysis from bitropyl. Ions and electrons are detected in coincidence, permitting us to record mass-selected photoelectron spectra. The threshold photoelectron spectrum of tropyl, corresponding to the *X*^+ 1^A_1_’ ← *X* ^2^E_2_” transition, reveals an ionisation energy of 6.23 ± 0.02 eV, in good agreement with Rydberg extrapolations, but slightly lower than the value derived from earlier photoelectron spectra. Several vibrations can be resolved and are reassigned to the C–C stretch mode ν_16_^+^ and to a combination of ν_16_^+^ with the ring breathing mode ν_2_^+^. Above 10.55 eV dissociative photoionisation of tropyl is observed, leading to the formation of C_5_H_5_^+^ and C_2_H_2_.

## Introduction

Organic radicals are known to be ubiquitous reactive intermediates in chemistry, biology and material science [1]. Studies on isolated radicals conducted in our group [2] yield their intrinsic properties, which are essential for understanding the reactivity of radicals in both the gas and condensed phase. Here we present a detailed study on the photoionisation of the cycloheptatrienyl radical (C_7_H_7_), commonly called tropyl, using synchrotron radiation.

**Figure 1:**
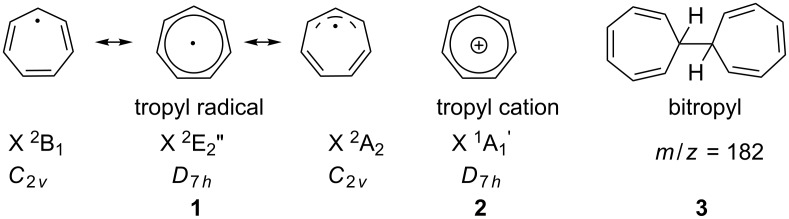
Structure of the tropyl radical **1**, its cation **2** and the precursor bitropyl **3**.

The tropyl radical **1** and its cation **2** are depicted in Figure 1. They have been at the focus of research since the 1960s [3–4] due to their symmetry properties. The interest originated in the expected stability of the tropyl cation, which is an aromatic molecular ion according to the Hückel rules. The aromaticity was confirmed and the symmetry of the C_7_H_7_^+^ established as *D*_7_*_h_* [5]. The vibrational structure of the cation was examined by IR and Raman spectroscopy [6–8]. There are 36 normal modes with 20 distinct frequencies, owing to degeneracy. Of these twenty vibrations, four are IR- and seven Raman-active [9].

In contrast to the cation, the odd-electron neutral tropyl radical is expected to be Jahn–Teller (JT) distorted. The nature of this distortion and whether the equilibrium structure of tropyl corresponds to a distorted *C*_2_*_v_* or to *D*_7_*_h_* symmetry has been studied experimentally and theoretically. An early electron spin resonance (ESR) experiment found seven equivalent hydrogen atoms and a uniform spin distribution and, therefore, concluded a dynamic *D*_7_*_h_* structure of the radical [10–11]. Calculations in the 1990s reported *C*_2_*_v_* symmetry due to Jahn–Teller distortion [9,12]. The most extensive investigation on the vibronic structure of the tropyl radical including the Jahn–Teller distortion was carried out by Miller and co-workers [13–14]. They found that the ^2^E_2_” ground state splits into two components of *C*_2_*_v_* symmetry, an allylic ^2^B_1_ and a dienylic ^2^A_2_ one (Figure 1). They are stabilized by roughly 1000 cm^−1^ with respect to the undistorted *D*_7_*_h_* saddle point [14], which corresponds to a conical intersection on the potential-energy surface. In addition to providing chemical insight, the other benefit of identifying the two *C*_2_*_v_* resonance geometries on the minimum path is that it makes geometry optimisations possible by the symmetry constraint. The IR spectrum of the radical was measured in the gas phase and compared to calculations as well as to that of the benzyl radical [15].

The geometry change upon ionisation and the character of the molecular orbitals triggered interest in the photoelectron spectroscopy of tropyl. The adiabatic ionisation energy of the radical was established by Thrush and Zwolenik (6.24 eV) [3], Elder and Parr (6.236 eV, derived from a photoion yield curve) [4] and Koenig and Chang (6.28 eV) [16]. The latter used helium(I) photoelectron spectroscopy and employed bitropyl **3** as a precursor (Figure 1). This molecule proved to be an efficient source for tropyl radicals generated by pyrolysis. Furthermore, the ground and excited states of the ion have been investigated computationally [9,17]. In the present study we extend the previous work using imaging photoelectron–photoion coincidence (iPEPICO) techniques in combination with VUV synchrotron radiation [18–20]. Coincidence spectroscopy correlates the electron signal with the mass signal and thus permits recording of mass-selected photoelectron spectra. This is particularly advantageous in experiments on reactive intermediates where a clean sample generation cannot always be ensured. An improved resolution is obtained from analysing only the threshold electrons [18,21], i.e., electrons recorded with almost zero initial kinetic energy upon tuning the photon energy. Thus, IR and Raman inactive ionic vibrations can often be observed and assigned in the photoelectron spectrum.

We have shown in the past that these techniques are well suited to study the photoionisation of open-shell species. Ionisation energies have been determined from vibrationally resolved photoelectron spectra for several open-shell species ranging from allyl [22] and propargyl [23] to indenyl (C_9_H_7_) [24], cyclopropenylidene [25] and fulvenallenyl [26]. In the case of allyl [27–28] and propargyl [29] they are in excellent agreement with high-resolution laser studies. Such data are important for the derivation of bond dissociation energies and heats of formation of radicals, but also aid in the in situ detection of radicals in flames by photoionisation [30]. The goal of the present experimental study was to elucidate the vibrational structure of the tropyl ion ground state with threshold photoelectron spectroscopic (TPES) techniques.

## Results and Discussion

**Figure 2:**
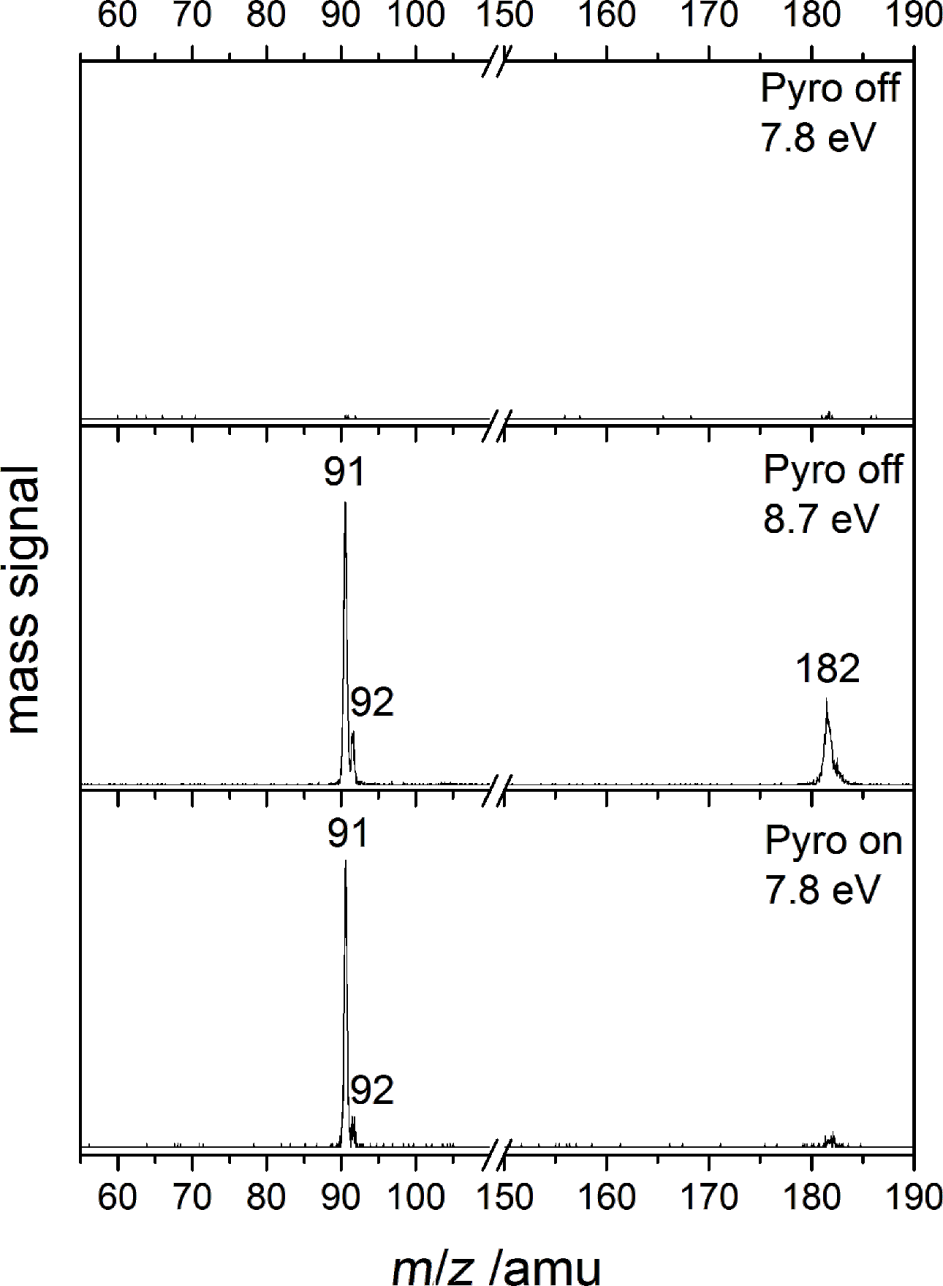
Mass spectra of bitropyl without pyrolysis at 7.8 and 8.7 eV (top and centre trace) and with pyrolysis, recorded at 7.8 eV (bottom trace).

The performance of the pyrolysis source can be illustrated by mass spectra at different photon energies with pyrolysis on or off, depicted in Figure 2.

As shown in the top trace of Figure 2 almost no signal is present without pyrolysis at a photon energy of 7.8 eV. At a photon energy of around 8 eV we start to see a signal of the bitropyl precursor **3**. Already at around the same energy a signal at *m*/*z* = 91 appears. Since there is no pyrolysis, the signal has to originate from dissociative photoionisation of bitropyl. Hence, in the mass spectrum recorded at 8.7 eV (centre trace of Figure 2) the observed masses are *m*/*z* = 91, 92 and 182, which correspond to a tropyl fragment, its ^13^C isotopologue and the bitropyl precursor. Above a photon energy of 8.9 eV, the *m*/*z* = 104 and 167 peaks appear in addition. They are products from higher energy dissociative photoionisation channels and are probably formed through the loss of benzene or a methyl group from the precursor, respectively. When we decrease the photon energy to 7.8 eV again and switch on the pyrolysis, (bottom trace in Figure 2) an intense photoionisation signal is observed at the masses *m*/*z* = 91 and 92, which is due to the direct photoionisation of tropyl and its ^13^C isotopologue. We note that the small signal at *m*/*z* = 182 never disappears completely. This is probably a result of sample contamination by an isomer of bitropyl. Thus, dissociative photoionisation may contribute to the tropyl signal at photon energies above 8 eV. Above 10.5 eV, an *m*/*z* = 65 peak appears exclusively in the “pyrolysis on” spectra corresponding to the dissociative photoionisation of the tropyl radical, yielding the cyclopentadienyl cation and acetylene.

**Figure 3:**
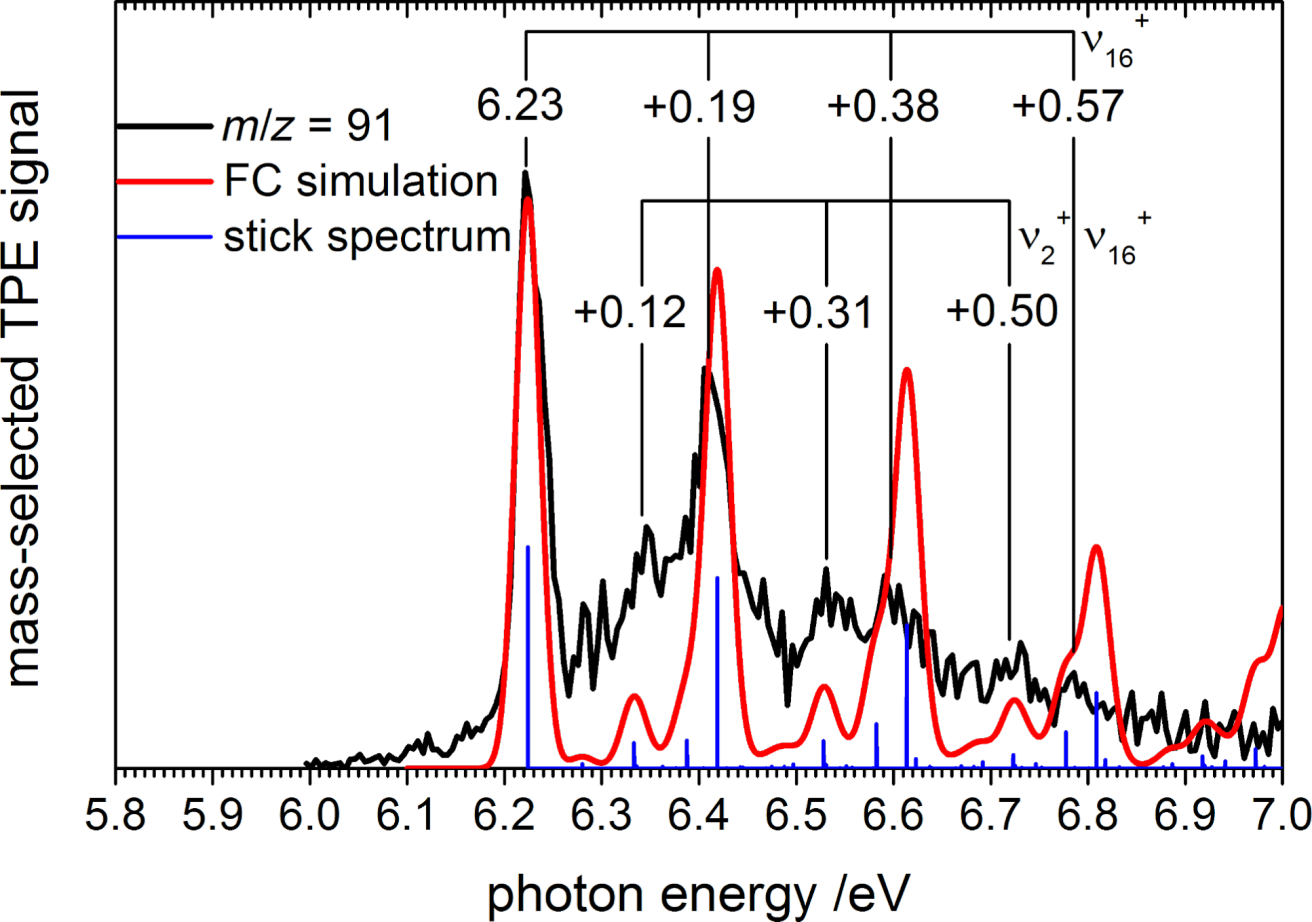
Threshold photoelectron spectrum of tropyl (black line). The Franck–Condon simulation (red line) is based on the computed stick spectrum (blue sticks) convoluted with 30 meV FWHM Gaussians.

**Figure 4:**
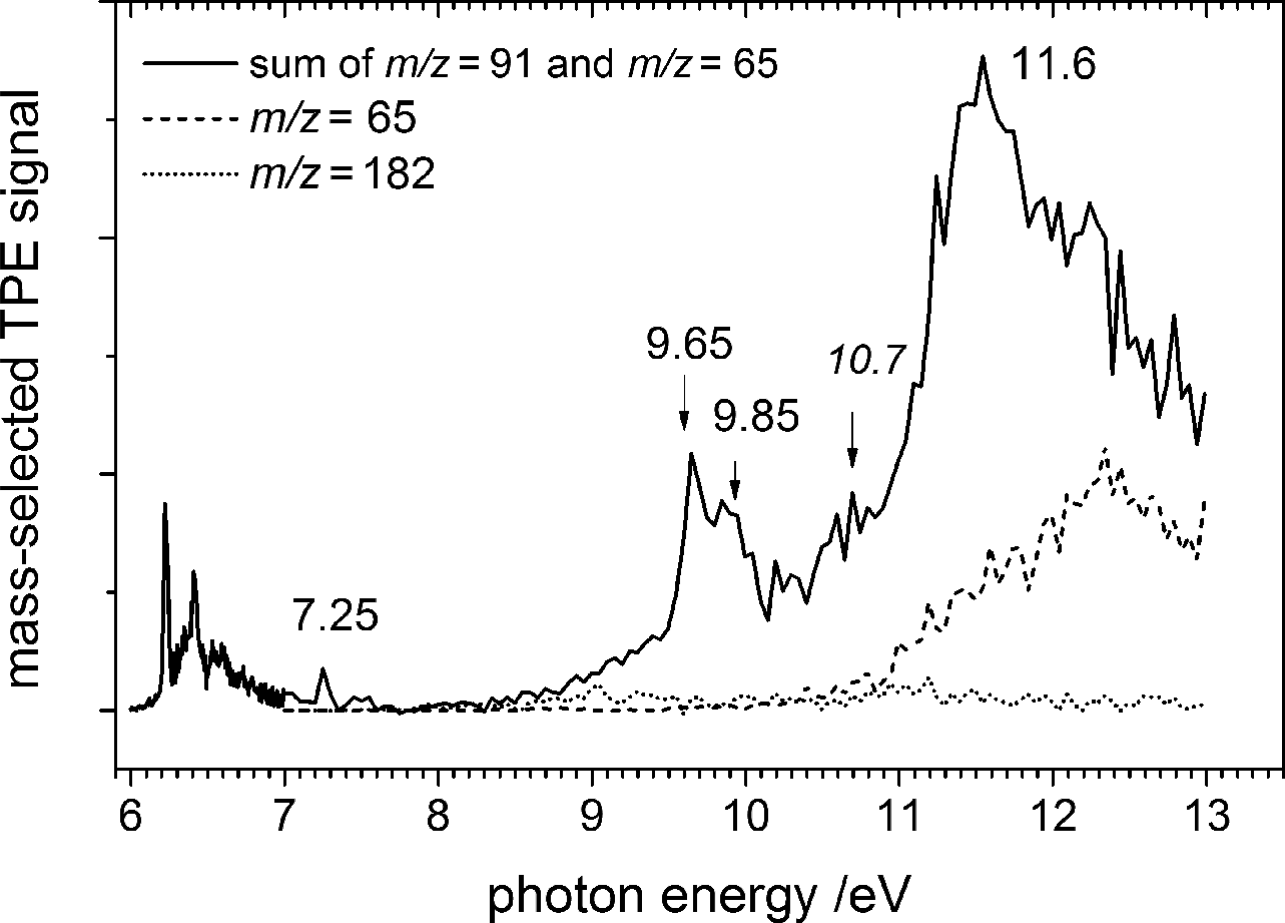
TPE spectrum of tropyl (solid line) and cyclopentadienyl (*m*/*z* = 65, dashed line) in the 7–13 eV photon energy range. Since C_5_H_5_^+^ is generated by dissociative photoionisation of tropyl at higher energies, the tropyl photoelectron spectrum is displayed as the sum of the two mass channels. The residual signal at the mass of the precursor is given as a dotted line for comparison.

Figure 3 shows the region of the ionisation onset of the tropyl radical in high resolution while Figure 4 (see below) exhibits the complete spectrum with the higher energy region in lower resolution. In both mass-selected threshold photoelectron (TPE) spectra the pyrolysis was turned on. The experimental spectrum (Figure 3) shows a sharp onset with a pronounced first maximum at 6.23 eV. It is assigned to the 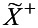 ^1^A_1_’ (v^+^=0) ← 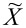 ^2^E_2_” (v” = 0) transition and corresponds to the adiabatic ionisation energy of the molecule. As the radical vibrational temperature is typically around 500 K in a continuous beam experiment [31] a contribution from hot and sequence bands cannot be excluded and could be responsible for the signal between 6.1 eV and 6.2 eV. The small peak at around 6.12 eV may correspond to a bending-mode hot band. Our IE value of 6.23 ± 0.02 eV is in excellent agreement with the values obtained from an extrapolation of Rydberg states: a [2 + 1] multiphoton ionisation (MPI) study [32] reported an IE of 50177 ± 46 cm^−1^ (6.221 eV) and the absorption experiment by Thrush and Zwolenik found a value of 6.24 eV. On the other hand, our value is slightly lower than the IE of 6.28 eV reported by conventional photoelectron spectroscopy [16]. It is interesting to note that the ionisation energy of benzyl, the second C_7_H_7_ isomer, lies at 7.249 eV [33] and is thus almost 1 eV higher.

A number of peaks are apparent that belong to vibrational progressions in the cation. The first progression has a spacing of around 1530 cm^−1^ (0.19 eV). This progression has been observed before. A vibrational spacing of 1528 ± 13 cm^−1^ was reported for high-lying Rydberg states and assigned to the overtone of a C–C–C bending mode of *e*_3_’ symmetry with a vibrational frequency of 768 cm^−1^ [32]. A vibrational progression with a spacing of 1424 ± 100 cm^−1^ was also found in conventional PES and attributed to a C–C–C stretching mode of *e*_1_ symmetry with a computed wavenumber of 1470 cm^−1^ [16]. A second pronounced peak is identified +0.12 eV above the origin (970 cm^−1^). This much weaker progression also appears in combination with members of the +0.19 eV progression, and has also been observed in the previous Rydberg state study [32]. Since it has now been firmly established that the minimum energy geometries are of *C*_2_*_v_* symmetry [13–14], these earlier assignments have to be reconsidered. Only totally symmetric modes, i.e., *a*_1_’ modes in the case of tropyl, appear as fundamentals in a photoelectron spectrum in the absence of vibronic coupling. However, upon photoionisation the symmetry of tropyl changes from *C*_2_*_v_* to *D*_7_*_h_*, so transitions have to be discussed in the common subgroup *C*_2_*_v_*. The irreducible representations *e*_1_’, *e*_2_’ and *e*_3_’ resolve into a_1_ 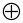 b_2_ upon going from *D*_7_*_h_* to *C*_2_*_v_*. As this sum contains the totally symmetric representation a_1_, these doubly degenerate modes that are symmetric towards σ_h_ are expected to be symmetry allowed in a *D*_7_*_h_* ← *C*_2_*_v_* transition.

In order to assign the vibrational transitions we performed a Franck–Condon (FC) simulation (Figure 3) with the FCfit program, version 2.8.8 [34] using the 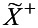 ^2^B_1_ allylic resonance structure of the neutral radical. We employed the geometry, frequencies and force constants of the CASSCF calculation from Stakhursky et al. [13] in the simulation. Note that their work accurately describes the Jahn–Teller distortion and thus represents the best available description of the radical potential-energy surface. They chose a (7,7) active space and employed a 6-31G(d) basis set. For the cation the input parameters were calculated by density functional theory (DFT) with the Gaussian 09 suite of programs [35], employing the B3LYP functional and a 6-31G(d) basis set. For a closed-shell molecule without vibronic distortions, a DFT approach provides the same accuracy for the geometries and frequencies as CASSCF. Our geometry and frequencies agree very well with the one reported in the literature [17]. For example a C–C bond length r(C–C) = 1.399 Å was found as compared to r(C–C) = 1.396 Å by Pino et al. [17]. For all vibrational frequencies unscaled values are given below. We note that the computations have been carried out in the Abelian point groups *C_s_* or *C*_2_*_v_*. The vibrational modes were assigned following the nomenclature of Lee and Wright [9], which has also been used by Pino et al. [17].

In order to compare the FC simulation with the experiment, the stick spectrum was convoluted with a Gaussian with a FWHM (full width at half maximum) of 0.030 eV. As seen in Figure 3, the simulation is in good agreement with the experimental spectrum. The main progression with a spacing of 1530 cm^−1^ (+0.19 eV) can be assigned to the doubly degenerate mode calculated at 1571 cm^−1^ (*e*_3_’, ν_16_^+^), which is an in plane C–C stretching vibration. Upon ionisation, the *D*_7_*_h_* saddle point turns into a true minimum in the absence of Jahn–Teller distortion. As this has to be associated with an adaption of the C–C bond lengths, the corresponding vibrations are expected to be active. In the experimental spectrum and the FC simulation we also observe its first (+0.38 eV, 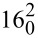) and second (+0.57 eV, 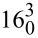) overtone. However, the simulation overestimates the intensity of the overtones. This is not surprising, considering that the neutral ground state geometry is delocalized and, in a crude approximation, we only use one resonance geometry and its harmonic oscillator functions. Also the second-order Jahn–Teller effect was neglected in the ground-state calculations. Still, no significant changes were observed in the simulated peak intensities when using the other *C*_2_*_v_* resonance geometry of the neutral, i.e., the dienylic ^2^A_2_ component, even though the geometries are somewhat different. The ground state can be described as a superposition of the ^2^A_2_ and ^2^B_1_ states, and it is reassuring that they both lead to very similar FC simulations. This also explains why the harmonic-oscillator approach works reasonably well in this case.

The first member of the second progression lies +0.12 eV above the origin and can be assigned to the ν_2_^+^ fundamental of *a*_1_’ symmetry in the reference geometry, 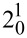. A wavenumber of 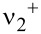 881 cm^–1^ was calculated for this ring breathing mode. This ν_2_^+^ mode has also been observed at +862 cm^−1^ (+0.11 eV) in the previous Rydberg state MPI study [32]. The band is not very pronounced and the maximum is difficult to identify in our spectrum. This probably explains the deviation to the computed value. Two further peaks are assigned to be combination bands with ν_16_^+^, namely 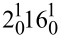 (+0.31 eV) and 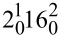 (+0.50 eV). An additional mode reported in the MPI spectrum at +1284 cm^–1^ is also predicted by the FC-simulations as visible in the stick spectrum in Figure 3. It is buried in the red edge of the +0.19 eV band. We assign it to the ν_17_^+^ C–H in-plane bend of e_3_’ symmetry, computed at 1320 cm^−1^.

Figure 4 presents the complete TPES up to 13.0 eV. Note that the photon energy step size changed at 7 eV. A small peak is observed at 7.25 eV. Most likely it corresponds to the adiabatic ionisation energy of the benzyl radical [36], originating either from precursor impurities or from an isomerisation in the pyrolysis. Benzyl is by about 70 kJ mol^−1^ more stable than tropyl [12,37–38], but a high activation barrier can be assumed for the isomerisation reaction in the pyrolysis source. Although we cannot exclude that the signal in the *m*/*z* = 91 mass channel might have some contributions from benzyl in the higher photon energy region, the benzyl signal is small compared to the tropyl one in the threshold region. Therefore the amount of the possible benzyl contamination is negligible.

**Figure 5:**
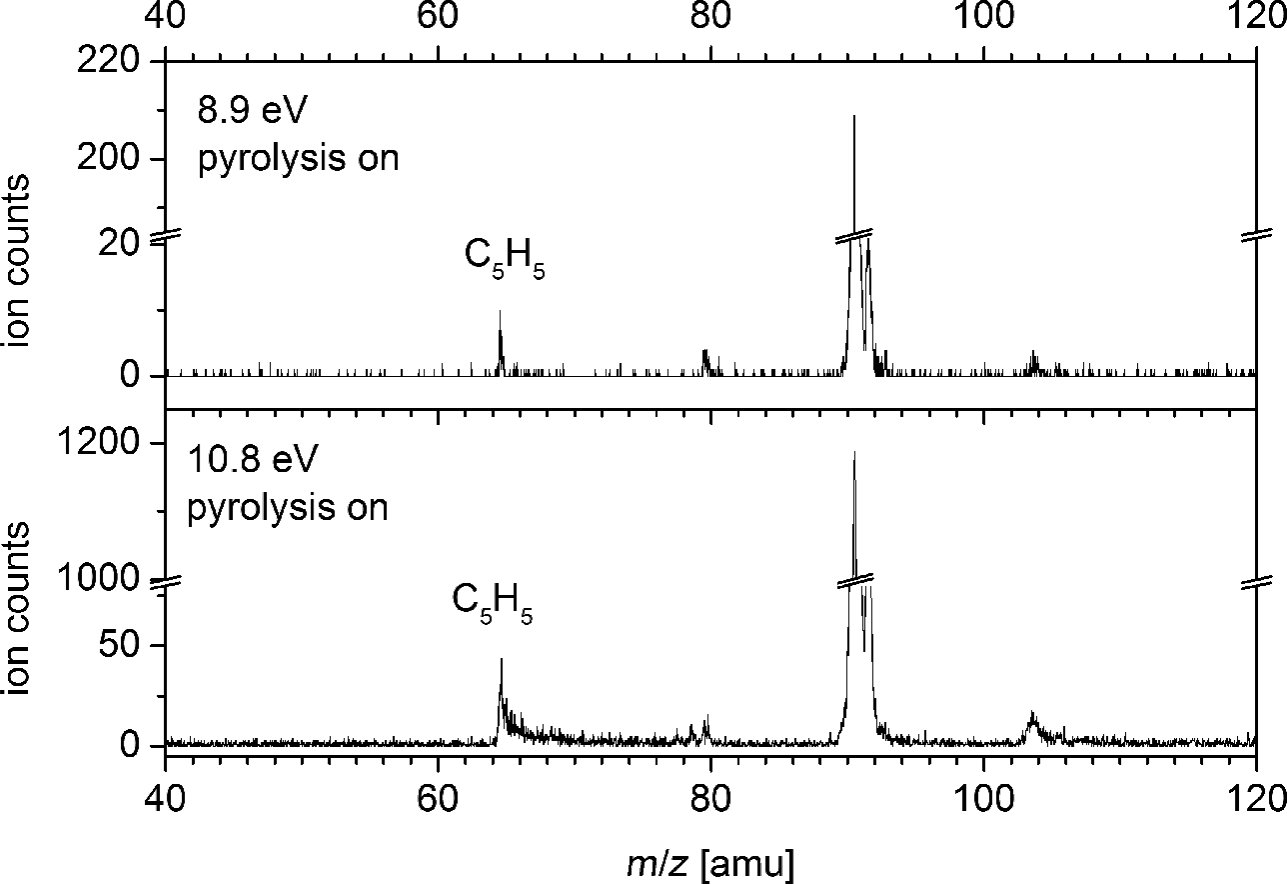
The shape of the C_5_H_5_^+^ peak in the mass spectrum changes with photon energy. While the peak is symmetric at 8.9 eV, it shows a pronounced asymmetry at 10.8 eV, indicating the onset of dissociative photoionisation.

With active pyrolysis an additional *m*/*z* = 65 peak appears at around 8.55 eV in the mass spectrum, which can be attributed to C_5_H_5_^+^. The mass-selected TPE-signal of *m*/*z* = 65 is depicted as a dashed line in Figure 4. Below 10.55 eV the signal is small and has a symmetric peak shape in the mass spectrum, as visible in the upper trace of Figure 5. Due to the small count number it is difficult to determine an accurate onset, but the signal appears around 8.55 eV. The adiabatic IE of the cyclopentadienyl radical was determined to be 8.428 eV by high-resolution photoelectron spectroscopy [39]. Thus, the appearance of C_5_H_5_^+^ in this energy range can be interpreted as the direct ionisation of cyclopentadienyl radical produced as a side product in the pyrolysis. Above 10.55 eV, on the other hand, the intensity rises significantly and the time-of-flight peak shape becomes asymmetric, as visible in the lower trace of Figure 5. Such an asymmetry indicates that the ion is a dissociation product of a metastable parent ion [40]. Formation of the cyclopentadienyl ion and acetylene upon dissociative photoionisation of tropyl can explain the rise in the mass channel above 10.55 eV and the asymmetric peak shape. Thermochemical calculations reveal that the channel is accessible at 10.52 eV, utilizing the heat of formation at 0 K of C_7_H_7_^+^ (896 kJ mol^−1^) [41], the ∆_f_*H*^o^ of cyclopentadienyl radical (276 kJ mol^−1^) [42], its adiabatic IE (8.428 eV = 813.18 kJ mol^−1^) [39], and the ∆_f_*H*^o^ of acetylene (226.88 kJ mol^−1^) [43]. This corresponds to around 4.3 eV internal energy in the ion before it dissociates, being in good agreement with our experimentally observed onset value of 10.55 eV. As pyrolysis is not complete, one has to consider dissociative photoionisation of the precursor as a possible source of C_5_H_5_^+^. The signal in the *m*/*z* = 182 mass channel, corresponding most likely to an isomer of bitropyl, is given as a dotted line in Figure 4. As can be seen, the signal is small throughout the studied energy range.

Between 7 eV and 8.5 eV the spectrum of tropyl has a Franck–Condon gap, and the transition to the first excited electronic state of the ion is visible at 9.65 eV. This peak has a shoulder at 9.85 eV photon energy, i.e., 200 meV higher, which may correspond to either vibrational excitation of the first electronic excited state or to the next electronic state. A noisy feature appears at 10.7 eV, and is followed by the highest intensity peak at 11.6 eV.

The most stable triplet state has been observed before at 9.63 eV [16], and is calculated to lie 3.1 eV above the ground state [9], i.e., at 9.33 eV using the newly determined adiabatic ionisation energy of 6.23 eV. In the same work, Lee and Wright predict the next triplet state at 3.9 eV excitation energy, i.e., at 10.13 eV photon energy. We calculated the EOM-CCSD/cc-pVTZ excitation energies for the triplet states, using QChem 4 [44] at the DFT-optimised ground state tropyl ion geometry, to be 3.82 and 4.00 eV, corresponding to 10.05 and 10.23 eV photon energies. TD-DFT calculations yield 9.70, 10.05 eV (B3LYP/6-311++G(d,p)); 9.86, 10.25 (M06-2X/6-311++G(d,p)) and 9.85, 10.03 eV (BLYP/6-311G(d,p)). Note that both triplet states are of E symmetry. The observed excitation energies are lower than the calculated values, which is due to possible Jahn–Teller distortions in the doubly degenerate triplet states, not considered in calculations of vertical excitation energies. The shoulder at 9.85 eV may thus either be due to the second triplet state, or to a vibrational fundamental of the excited state. For example a C–C stretching mode may be responsible for this peak, 1600 cm^–1^ further to the blue with respect to the first one.

The electronic spectroscopy of mass-selected tropyl cations has recently been investigated in Ne matrix [45]. A progression starting at 275.1 nm (4.51 eV) was assigned to the ^1^A_2_” ←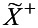 ^1^A_1_’ transition of the tropyl cation, corresponding to 10.74 eV in the photoelectron spectrum for the first excited singlet state of the ion. The shoulder at around 10.7 eV in Figure 4 might be tentatively assigned to this state. However, previous calculations predicted the first singlet excited state at photon energies of 11.3 [9] and 11.74 eV [45]. Our EOM-CCSD result at 11.07 and TD-DFT results at 11.09, 11.24 and 10.95 eV with the B3LYP, M06-2X and BLYP functionals, respectively, agree reasonably well with the experimental result of Nagy et al. [45].

Overall the theoretical predictions are less consistent in the range of the maximum TPE signal at 11.6 eV (Figure 4). Lee and Wright reported a further triplet state at 12.4 eV, followed by a 1 eV gap to the next electronic excited state. EOM-CCSD vertical-excitation-energy calculations predict that the next higher lying state is a triplet at 12.48 eV followed by two more triplet states in ca. 100 meV intervals as well as several singlet states around 12.8 eV. TD-DFT calculations, on the other hand, depend greatly on the functional used. B3LYP calculations agree best with the experiment, yielding both a singlet and a triplet state in the 11.8–11.9 eV photon energy range. BLYP yields almost a continuum of states at 11.38 (S), 11.45 (T), 11.54 (S), 11.60 (S) and 11.66 (S) eV, whereas the next singlet state above 11.24 eV is obtained at 12.05 eV with the M06-2X functional. To summarize, wave-function methods, such as CIS, employed by Lee and Wright or EOM-CCSD predict a sparse electronic excitation spectrum with a gap at the experimentally observed main peak at 11.6 eV. Density functional results, on the other hand, are inconsistent in this energy range. Thus an unequivocal assignment of this band is difficult.

## Conclusion

We studied the photoionisation of the tropyl radical, generated by pyrolysis of bitropyl, employing the iPEPICO technique. The first band in the mass-resolved threshold photoelectron spectrum at 6.23 eV was assigned to the adiabatic ionisation energy. This value is in very good agreement with a previous extrapolation of Rydberg states. With the help of a Franck–Condon simulation two progressions were assigned. The first includes the vibration ν_16_^+^, an *e*_3_’ C–C stretching mode with a spacing of 1530 cm^−1^ (0.19 eV), while the second progression is a combination of the ν_2_^+^ *a*_1_’ ring-breathing mode and ν_16_^+^. The simulations also indicate activity in the ν_17_^+^ C–H in-plane bending mode of *e*_3_’ symmetry. Moreover the first triplet and (possibly) singlet excited states of the tropyl ion were observed at 9.65 eV and 10.7 eV, respectively, in agreement with earlier work [5,16]. The second triplet state may also be visible at 9.85 eV. The most intense band appears at 11.6 eV. Computing this part of the spectrum proved challenging, with wave-function methods predicting a gap in this energy range, while DFT results depend greatly on the functional used. At around 10.55 eV (4.3 eV internal energy) the tropyl ion starts to photoionize dissociatively to form the cyclopentadienyl ion. This value is in very good agreement with the appearance energy estimated from a thermochemical cycle.

## Experimental

The experiments were carried out at the VUV beamline of the Swiss Light Source at the Paul Scherrer Institut (PSI) in Villigen, Switzerland. The beamline has been described in detail elsewhere [46–47]. The X04DB bending magnet provides synchrotron radiation, which is collimated and sent to a plane grating monochromator with a 600 grooves/mm with a maximum resolving power of 10^4^. A mixture of 10% Kr, 30% Ar and 60% Ne at a pressure of 10 mbar was used to suppress radiation at higher harmonics in a differentially pumped gas filter. Below 7 eV a MgF_2_ window was used instead of the gas mixture. A photon energy resolution of 5 meV was achieved at 15.764 eV, measured at the 11s resonance of argon.

The iPEPICO (imaging photoelectron photoion coincidence) technique was employed to study the photoionisation of tropyl **1**. This technique allows the mass-selective detection of threshold photoelectrons by detecting them in coincidence with ions. The spectrometer is a combination of a Wiley–McLaren time-of-flight (TOF) mass spectrometer [48] and a velocity map imaging setup [49]. The latter is equipped with a position sensitive detector with a delay line anode (Roentdek DLD40). Only the central part of the electron image, corresponding to an electron energy resolution of around 5 meV, was taken for further analysis. The contribution of hot background electrons was subtracted following the method outlined by Sztáray and Baer [50].

A flange equipped with a molecular beam source and a SiC tube for flash pyrolysis [51] was mounted to the vacuum chamber. Bitropyl **3** was synthesized according to the literature [5], placed in an oven and heated to 90–105 °C to obtain a sufficient vapour pressure. The precursor was seeded in an argon flow of around 70 mbar and expanded through a 0.1 mm pinhole into the pyrolysis tube. The oven was mounted in line with the gas flow. An unskimmed jet was employed. The photon energy was scanned in steps of 5 meV in the region of the ionisation threshold and 10–50 meV in the higher energy regions. Data were averaged for 60 seconds per data point.
